# Inadequate treatment of ventilator-associated and hospital-acquired pneumonia: Risk factors and impact on outcomes

**DOI:** 10.1186/1471-2334-12-268

**Published:** 2012-10-24

**Authors:** Nihal Piskin, Hande Aydemir, Nefise Oztoprak, Deniz Akduman, Fusun Comert, Furuzan Kokturk, Guven Celebi

**Affiliations:** 1 Departments of Infectious Diseases and Clinical Microbiology, School of Medicine, Zonguldak Bulent Ecevit University, Zonguldak, Turkey; 2 Department of Infectious Diseases and Clinical Microbiology, Antalya Teaching and Research Hospital, Antalya, Turkey; 3 Department of Microbiology and Clinical Microbiology, Zonguldak Bulent Ecevit University, Zonguldak, Turkey; 4 Deparment of Biostatistics, Zonguldak Bulent Ecevit University, Zonguldak, Turkey

## Abstract

**Background:**

Initial antimicrobial therapy (AB) is an important determinant of clinical outcome in patients with severe infections as pneumonia, however well-conducted studies regarding prognostic impact of inadequate initial AB in patients who are not undergoing mechanical ventilation (MV) are lacking. In this study we aimed to identify the risk factors for inadequate initial AB and to determine its subsequent impact on outcomes in both ventilator associated pneumonia (VAP) and hospital acquired pneumonia (HAP).

**Methods:**

We retrospectively studied the accuracy of initial AB in patients with pneumonia in a university hospital in Turkey. A total of 218 patients with HAP and 130 patients with VAP were included. For each patient clinical, radiological and microbiological data were collected. Stepwise multivariate logistic regression analysis was used for risk factor analysis. Survival analysis was performed by using Kaplan-Meier method with Log-rank test.

**Results:**

Sixty six percent of patients in VAP group and 41.3% of patients in HAP group received inadequate initial AB. Multiple logistic regression analysis revealed that the risk factors for inadequate initial AB in HAP patients were; late-onset HAP (OR = 2.35 (95% CI, 1.05-5.22; p = 0.037) and APACHE II score at onset of HAP (OR = 1.06 (95% CI, 1.01-1.12); p = 0.018). In VAP patients; antibiotic usage in the previous three months (OR = 3.16 (95% CI, 1.27-7.81); p = 0.013) and admission to a surgical unit (OR = 2.9 (95% CI, 1.17-7.19); p = 0.022) were found to be independent risk factors for inadequate initial AB. No statistically significant difference in crude hospital mortality and 28-day mortality was observed between the treatment groups in both VAP and HAP. However we showed a significant increase in length of hospital stay, duration of mechanical ventilation and a prolonged clinical resolution in the inadequate AB group in both VAP and HAP.

**Conclusion:**

Our data suggests that the risk factors for inadequate initial AB are indirectly associated with the acquisition of resistant bacteria for both VAP and HAP. Although we could not find a positive correlation between adequate initial AB and survival; empirical AB with a broad spectrum should be initiated promptly to improve secondary outcomes.

## Background

Nosocomial pneumonia, which is usually defined as hospital-acquired pneumonia (HAP), is the second most frequent nosocomial infection but the first in terms of morbidity, mortality and cost. It occurs in 8-20% of intensive care unit (ICU) patients, with an increased frequency and mortality if the patients are mechanically ventilated [1-4]. Over the past decade, several risk factors associated with mortality have been detected in HAP and ventilator-associated pneumonia (VAP). One of the most consistent and evident prognostic factor throughout the literature is the accuracy of initial antibiotic treatment (AB). By contrast, early aggressive therapy with adequate broad-spectrum regimens that optimize therapy against the likely pathogens is associated with lower mortality rates and shorter hospital stay [4-8]. However, despite numerious studies some controversies continue to exist about the genuine prognostic impact of initial AB [9-14].

In recent years, international societies and most recently, the American Thoracic Society jointly with the Infectious Diseases Society of America, have developed guidelines for the management of HAP and VAP [2,3]. In spite of the presence of practice guidelines, the percentage of inadequate AB varies in the literature from 10% to 73% [8-11,13-18]. The presence of multidrug-resistant bacteria is the primary reason that patients with VAP and HAP receive inadequate AB [5], however other factors that contribute to inadequate AB are not well defined. Therefore, we aimed to identify the possible risk factors for inadequate initial AB in both HAP and VAP and to determine its subsequent impact on outcomes.

## Methods

### Patients and setting

This retrospective cohort study was conducted at Zonguldak Bulent Ecevit University Hospital, a 524 bed referral and tertiary hospital, from January 2005 to January 2008. Patients were enrolled in the study if they were >16 years of age and diagnosed with VAP or HAP irrespective of the diagnosis at admission. Only the first episode of pneumonia was taken into account and no community acquired pneumonia cases were included. The study was approved by the Zonguldak Bulent Ecevit University Hospital Ethics Committee. The need for informed consent was waived due to the retrospective observational nature of the study.

For each patient clinical, radiological and microbiological data were collected. The following characteristics were retrieved from patient records: age, sex, underlying diseases, antibiotic usage in the previous three months, Acute Physiological Score Chronic Health Evaluation (APACHE) II at onset of pneumonia, length of hospital and ICU stays, presence of invasive procedures, duration of mechanical ventilation, presence of an other site infection, microbiological culture results, fever and/or other clinical symptoms resolution dates and mortality.

### Definitions

Hospital-acquired pneumonia was diagnosed on the basis of Centers for Disease Control and Prevention clinical criteria [19]. Pneumonia was considered ventilator associated when it occurred 48 hours after starting mechanical ventilation and it was known to have not been incubating before the initiation of MV. Pneumonia was defined as early-onset if it started within 4 days of admission, in accordance with the American Thoracic Society/Infectious Disease Society of America guidelines [2,3]. Quantitative cultures of respiratory samples obtained at the time of diagnosis were used to diagnose pneumonia. Inadequate AB was defined as, at least one bacterial isolate not covered by any initial antibiotic, or when the pathogen was resistant to all initial empiric antimicrobial agents. In culture negative cases, patients’ records were evaluated and appropriateness of AB was decided according to resolution of clinical and laboratory signs of pneumonia with the initial regimen that had been pursued or changed. AB was modified as soon as susceptibility testing results were available or if the clinical and radiological signs of pneumonia did not improve, mainly within 48–72 hours. The total number of deaths within 28 days after the onset of pneumonia and during hospitalization were defined as 28-day mortality and in-hospital mortality respectively.

### Microbiological data

Microbiological data for the patients was obtained from cultures of sputum, transendotracheal aspirates (TA), blood, pleural fluids and bronchoalveolar lavage (BAL) fluids. The bacteriological diagnosis required the following criteria: Sputum and TA cultures growing ≥ 10^5^ colony-forming units (cfu)/ml of bacteria, BAL cultures growing 10^4^ cfu/ml of bacteria, blood or pleural fluid cultures revealing the same pathogen with the respiratory samples. Appropriate specimens of sputum and TA to be taken into consideration for diagnostic testing required a white blood cell count of >25 and <10 epithelial cells per low-power examination field. Bacterial identification and susceptibility testing were performed by standard methods. The identification of “mouth flora” was considered as culture negative.

### Statistical analysis

Statistical analysis was performed with SPSS 18.0 software (SPSS, Inc., Chicago, IL, USA). Continuous variables were expressed as mean ± standard deviation and categorical variables as numbers and percentages. Continuous variables were compared with the Independent Sample T test or Mann–Whitney *U* test and categorical variables were compared using Pearson's Chi-square test or Fisher’s Exact Chi-square test. A *P* value of less than 0.05 was considered statistically significant for all tests. Forward stepwise logistic regression model was performed to assess the independent risk factors for inadequate initial antibiotic treatment in both HAP and VAP patients. Survival analysis was performed using Kaplan-Meier method with Log-rank test.

## Results

During the study period 348 patients met the inclusion criteria in whom 218 patients developed HAP and 130 patients developed VAP. The mean age of the patients was 63.56 ± 15.17 and 62.9% were male. The most frequent admission diagnoses included respiratory diseases (namely COPD, ARDS or acute respiratory failure) (32.3%), neurological diseases (17.7%), malignancy (13.1%) in VAP patients and respiratory diseases (34.9%), malignancy (20.2%) and metabolic diseases (27.1%) in HAP patients. Eighty six (66%) patients in VAP group and 90 (41.3%) patients in HAP group received inadequate initial AB. Characteristics of patients with HAP and VAP with regard to the adequacy of empirical AB are summarized in Tables 1 and 2 respectively. Age, sex, comorbidities and admission diagnosis were not statistically different between treatment groups in either HAP or VAP patients.

**Table 1:** Characteristics of 218 patients with HAP with regard to the adequacy of empirical antibiotic treatment

**Characteristics**	**Adequate treatment n = 128**	**Inadequate treatment n = 90**	**p**
Age (years)	61.84 ± 14.66	64.44 ± 13.49	0.189
Sex (male)	84 (65.6)	58 (64.4)	0.857
Surgical unit	24 (68.6)	11 (31.4)	0.269
Underlying diseases			
Diabetes mellitus	26 (20.3)	14 (15.6)	0.474
COPD	32 (25.0)	19 (21.1)	0.613
Chronic renal failure	14 (10.9)	10 (11.1)	1.000
Congestive heart failure	15 (11.7)	16 (17.8)	0.287
Cerebrovascular disease	7 (5.5)	10 (11.1)	0.203
Malignancy	24 (18.8)	22 (24.4)	0.398
Central venous catheterization	37 (28.9)	35 (38.9)	0.123
Urinary catheterization	88 (68.8)	66 (73.3)	0.464
Late-onset pneumonia	78 (60.9)	72 (80.0)	**0.003**
Acquisition of other site infection	21 (16.4)	20 (22.2)	0.365
Previous antibiotic usage	42 (32.8)	34 (37.8)	0.449
Culture proven pneumonia	43 (33.6)	51 (56.7)	**0.001**
MDR bacteria	29 (22.7)	38 (42.2)	**0.002**
Polymicrobial etiology	1 (0.8)	8 (8.9)	**0.004**
Length of stay before HAP	7.09 ± 5.07	8.52 ± 5.26	**0.003**
APACHE II score	12.68 ± 5.79	14.63 ± 6.59	**0.024**

**Table 2:** Characteristics of 130 patients with VAP with regard to the adequacy of empirical antibiotic treatment

**Characteristics**	**Adequate treatment n = 44**	**Inadequate treatment n = 86**	**p**
Age (years)	65.64 ± 16.42	64.15 ± 16.83	0.628
Sex (male)	29 (65.9)	48 (55.8)	0.358
Surgical unit	22 (45.8)	26 (54.2)	**0.044**
Underlying diseases			
Diabetes mellitus	6 (13.6)	13 (15.1)	1.000
COPD	15(34.1)	18 (20.9)	0.156
Chronic renal failure	1 (2.3)	4 (4.7)	0.672
Congestive heart failure	7 (15.9)	15 (17.4)	1.000
Cerebrovascular disease	5 (11.4)	16 (18.6)	0.418
Malignancy	6 (13.6)	13 (15.1)	1.000
Central venous catheterization	34 (77.3)	62 (72.1)	0.671
Urinary catheterization	43 (97.7)	84 (97.7)	1.000
Late-onset VAP	36 ( 81.8)	75 (87.2)	0.575
Presence of other site infection	12 (27.3)	42 (48.8)	**0.030**
Previous antibiotic usage	12 (27.3)	39 (45.3)	0.071
Culture proven pneumonia	37 (84.1)	80 (93.0)	0.128
Polymicrobial etiology	2 (4.5)	6 (7.0)	0.186
MDR bacteria	32 (72.7)	60 (69.8)	0.883
Length of stay before VAP	12.61 ± 11.20	14.31 ± 10.348	0.138
APACHE II score	16.39 ± 6.71	16.81 ± 6.63	0.730

In all VAP cases TA and/or BAL cultures were performed. While for the HAP group, TA cultures were performed in 45 patients and BAL cultures were performed in 7 patients, the remaining samples were all sputum cultures. Of the 130 patients with VAP, 13 (10%) were culture negative, eight patients (6.1%) had polymicrobial infection and 92 (78.6%) patients were infected by a multi-drug resistant pathogen. The number of cultures which grew non-fermenting gram-negative bacilli was higher in the inadequate AB group but the difference was not statistically significant (Table 3). Of the 218 patients with HAP, 124 (56.9%) were culture negative, nine patients (4.1%) had polymicrobial infection and 67 (30.7%) patients were infected by a multi-drug resistant pathogen. Totally 103 pathogens were isolated from 94 patients. The isolated pathogens are presented in Table 3.

**Table 3:** Microorganisms recovered from patients with HAP and VAP

**Bacteria**		HAP (n = 103)*			VAP (n = 125)*	
	**Adequate n = 49**	**Inadequate n = 54**	**p**	**Adequate n = 41**	**Inadequate n = 84**	**p**
Acinetobacter spp	8 (7.8)	13 (12.6)	0.499	13 (10.4)	37 (29.6)	0.383
Pseudomonas spp	10 (9.7)	14 (13.6)		9 (7.2)	21 (16.8)	
S. aureus	12 (11.6)	7 (6.8)		10 (8)	13 (10.4)	
S.pneumoniae	6 (5.8)	5 (4.8)		1 (0.8)	5 (4)	
K. pneumonia	3 (2.9)	3 (2.9)		3 (2.4)	2 (1.6)	
E.coli	6 (5.8)	5 (4.8)		2 (1.6)	4 (3.2)	
Others	4 (3.9)	7 (6.8)		3 (2.4)	2 (1.6)	

n = Number of microorganisms in patient groups.

The regimens of empiric antibiotics are summarized in Table 4. Thirty three (25.4%) patients in VAP group and 13 (5.9%) patients in HAP group received an antibiotic combination with a glycopeptide antibiotic. Among the patients who received inadequate initial AB, antibiotic therapy was modified 4.84 ± 2.49 and 3.80 ± 2.49 days after the onset of pneumonia in VAP and HAP patients respectively.

**Table 4:** Antimicrobial agents used in patients with HAP and VAP

**Empiric antibiotic**		**HAP (n = 218)**			**VAP (n = 130)**	
	**Adequate (n = 128)**	**Inadequate (n = 90)**	**p**	**Adequate (n = 44)**	**Inadequate (n = 86)**	**p**
Carbapenems	31 (24.2)	16 (17.8)	0.004	20 (45.5)	18 (20.9)	<0.001
Third generation cefalosporine	31 (24.2)	27 (30.0)		4 (9.1)	20 (23.3)	
β-lactam + β- lactamase inhibitor	35 (27.3)	21 (23.3)		14 (31.8)	26 (30.2)	
Quinolone	21 (16.4)	16 (17.8)		0	10 (11.6)	
β-lactam in combination with quinolone	1 (0.8)	9 (10.0)		0	9 (10.5)	
Aminoglycoside in combination with carbapenem or β-lactam	9 (7.0)	1 (1.1)		6 (13.6)	3 (3.5)	

As the number of culture negative pneumonias were high especially in the HAP group, we also performed the analysis in “culture positive patients only” in both HAP and VAP to determine whether the risk factors differed based on culture positivity. The results are presented in Table 5.

**Table 5:** Characteristics of culture positive patients with HAP and VAP with regard to the adequacy of empirical antibiotic treatment

**Characteristics**		**HAP (n = 94)**			**VAP (n = 117)**	
	**Adequate treatment n = 43 (%)**	**Inadequate treatment n = 51 (%)**	**p**	**Adequate treatment n = 37 (%)**	**Inadequate treatment n = 80 (%)**	**p**
Age (years)	60.67 ± 16.04	65.14 ± 13.75	0.167	63.59 ± 16.65	63.85 ± 17.07	0.907
Sex (male)	32(74.4)	37 (72.5)	1.000	24 (64.9)	45 (56.3)	0.497
Surgical unit	9 (20.9)	8 (15.7)	0.697	21 (56.8)	25 (31.3)	**0.015**
Underlying diseases						
Diabetes mellitus	5 (11.6)	8 (15.7)	0.789	5 (13.5)	11 (13.8)	1.000
COPD	13 ( 30.2)	10 (19.6)	0.341	11 (29.7)	17 (21.3)	0.443
Chronic renal failure	5 (11.6)	4 (7.8)	0.727	0 (0)	3 (3.8)	0.551
Congestive heart failure	4 (9.3)	9 (17.6)	0.386	6 (16.2)	13 (16.3)	1.000
Cerebrovascular disease	1 (2.3)	9 (17.6)	**0.019**	3 (8.1)	15 (18.8)	0.227
Malignancy	6 (14.0)	9 (17.6)	0.838	6 (16.2)	11 (13.8)	0.944
Central venous catheterization	16 (37.2)	22 (43.1)	0.710	30 (81.1)	58 (72.5)	0.442
Urinary catheterization	33 (76.7)	37 (72.5)	0.820	37 (100)	78 (97.5)	1.000
Late-onset pneumonia	34 (79.1)	46 (90.2)	0.223	34 (91.9)	72 (90)	1.000
Acquisition of other site infection	12 (27.9)	11 (21.6)	0.637	11 (29.7)	42 (52.5)	**0.036**
Previous antibiotic usage	20 (46.5)	23 (45.1)	1.000	11 (29.7)	36 (45.0)	0.173
MDR bacteria	29 (67.4)	38 (74.5)	0.599	32 (86.5)	60 (75)	0.243
Polymicrobial etiology	1 (2.3)	8 (15.7)	**0.036**	2 (5.4)	6 (7.5)	1.000
Length of stay before HAP	7.6 ± 4.3	9.18 ± 5.8	0.194	13.89 ± 11.56	14.88 ± 10.34	0.313
APACHE II score	12.7 ± 5.83	14.92 ± 5.83	**0.031**	16.08 ± 6.94	16.69 ± 6.72	0.654
Exitus	16 (37.2)	24 (47.1)	0.452	27 (73)	53 (66.3)	0.608

To define the independent risk factors for inadequate initial AB in both HAP and VAP; multiple logistic regression analysis was performed and age, gender, type of ICU and/or general ward, presence of underlying diseases, presence of central venous catheterization and mechanical ventilation, duration of intubation (for VAP patients only), duration of hospitalization before onset of pneumonia, previous antibiotic usage in the previous three months, APACHE II score at onset of pneumonia, diagnosis of pneumonia (late-onset) and presence of multi-drug resistant bacteria were included to the model. There were no multicolinearity in the variables included in the regression model. Multiple logistic regression revealed that the risk of inadequate initial AB in HAP patients was more than twice as large among patients with late-onset HAP (OR = 2.35 (95% CI, 1.05-5.22; p = 0.037) and the risk was also found to be significantly associated with the higher APACHE II score at the onset of HAP (OR = 1.06 (95% CI, 1.01-1.12); p = 0.018). In VAP patients; antibiotic usage in the previous three months (OR = 3.16 (95% CI, 1.27-7.81); p = 0.013) and admission to a surgical unit (OR = 2.9 (95% CI, 1.17-7.19); p = 0.022) were found to be independent risk factors for inadequate initial AB.

**Figure 1:**
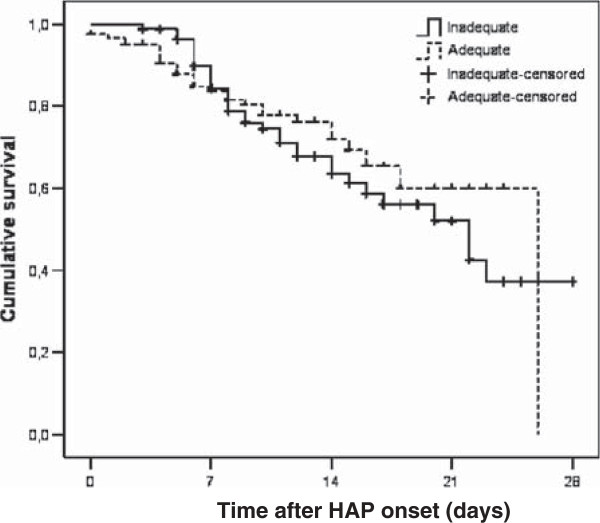
Kaplan-Meier analysis of empirical antimicrobial treatment of HAP according to 28-day mortality (p = 0.519) log rank test.

**Figure 2:**
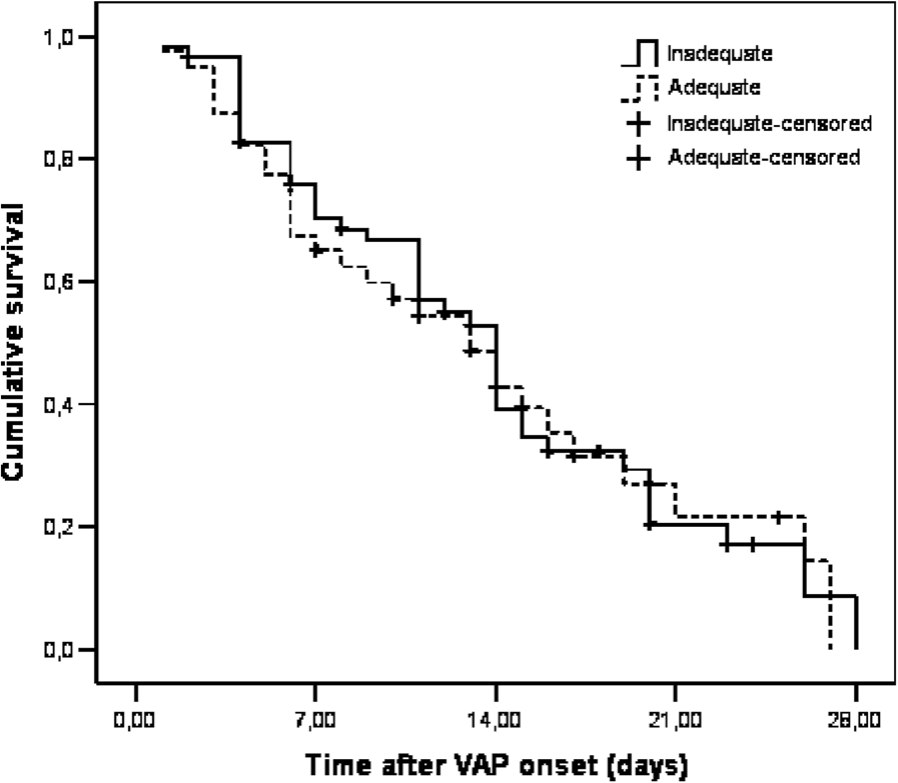
Kaplan-Meier analysis of empirical antimicrobial treatment of VAP according to 28-day mortality (p = 0.847) log rank test.

Sixty eight (31.2%) patients out of 218 died in the HAP group and 88 (67.7%) patients died in the VAP group. In the HAP group 95 (43.6%) patients were admitted to an ICU during their hospital stay and 54 (56.8%) of these died. The remaining 123 patients were followed in other wards and 14 (11.4%) of these died. The difference was statistically significant (p < 0.001). The outcome measures of patients who did and did not receive adequate initial AB are summarized in Table 6. No statistically significant difference in crude hospital mortality was observed between the groups in both VAP and HAP (Table 6). The pneumonia related mortality also did not reach statistical significance by day 28 after the diagnosis of pneumonia in either HAP or VAP [25.2% (30/119) with adequate initial AB versus 39.5% (32/81) with inadequate initial AB in HAP patients (p = 0.519) (Figure 1) and 75.0% (30/40) with adequate initial AB versus 71.2% (42/59) with inadequate initial AB in VAP patients (p = 0.847) (Figure 2)]. In VAP patients median survival time after VAP onset was 13 days with adequate initial AB and 14 days with inadequate initial AB (Figure 1). In HAP patients median survival time after HAP onset was 26 days with adequate initial AB and 22 days with inadequate initial AB (Figure 2).

**Table 6:** Outcome of patients who did and did not receive adequate antimicrobial therapy

		**HAP (n = 218)**			**VAP (n = 130)**	
	**Adequate (n = 128)**	**Inadequate (n = 90)**	**p**	**Adequate (n = 44)**	**Inadequate (n = 86)**	**p**
In hospital mortality (n,%)	32 (25.8)	35 (38.9)	0.056	32 (72.7)	56 (65.1)	0.497
Length of total hospital stay	19.61 ± 14.40	24.22 ± 13.70	**0.001**	28.57 ± 23.66	45.00 ± 49.30	**0.009**1
Length of stay after the diagnosis of pneumonia	12.52 ± 11.14	15.70 ± 11.21	**0.010**	16.45 ± 18.23	30.71 ± 46.21	**0.034**
Duration of intubation				19.11 ± 19.74	32.14 ± 49.51	**0.050**
Resolution of fever and other symptoms	2.40 ± 2.91	4.96 ± 7.79	**<0.001**	5.70 ± 5.50	9.09 ± 10.33	**0.044**

## Discussion

Despite numerious studies in the literature, most of our knowledge about the risk factors and prognostic impact of inadequate initial AB was based on studies of VAP because well-conducted studies involving patients who are not undergoing mechanical ventilation are lacking. Similarly the American Thoracic Society/Infectious Diseases Society of America evidence-based guidelines for nosocomial pneumonia are mainly based on studies of VAP but it is recommended to treat patients who were not intubated or undergoing mechanical ventilation, in the same manner as those with VAP [3]. Although treatment alternatives are similar, in our study we planned to evaluate the risk factors for inadequate initial AB and the impact of adequacy of initial AB on the prognosis of patients with HAP and VAP separately.

In this study 66.6% of patients in VAP and 41.3% of patients in HAP received inadequate initial AB. The percentage of patients who received inadequate initial AB is quite variable in the previously published studies and changes from 10% to 73% [8-11,13-18]. The knowledge regarding the colonization of patients, the need to limit selection pressure and the sensitivity profile of the suspected pathogens may influence the frequency of inappropriateness of initial AB.

We identified higher APACHE II score and late-onset pneumonia as independent risk factors for inadequate initial AB for patients with HAP. Although severity indices are not always mentioned in studies regarding the adequacy of AB of pneumonia, patients with higher severity are considered to be at high risk of poor outcome and they are also likely to be infected by more resistant bacteria [10,11]. Similarly late-onset nosocomial pneumonia is caused most frequently by hospital-acquired and often MDR pathogens [20-22]. The potential involvement of more resistant bacteria in the late-onset pneumonia is more likely to lead to inadequate treatment with traditional antibiotic regimens [4,6,8,22]. However, we did not find such association in late onset pneumonia regarding the adequacy of initial AB and susceptibility patterns of the isolated pathogens when we performed the analysis in culture positive patients only. This conflicting result may be explained by the distribution of MDR bacteria within the groups. In our study cohort the presence of MDR bacteria was higher in the inadequate treatment arm in HAP patients and this was statistically significant. But this significance could not be shown when only culture positive HAP patients were analyzed. So we may conclude that the causative pathogens in late-onset pneumonia in culture negative HAP patients were resistant to the initial empiric AB. Although the presence of polymicrobial etiology were found to be associated with inadequate initial AB in HAP patients both in general and in the subgroup analysis of the culture positive patients, it was not determined as an independent risk factor. We believe that this may be due to the small number of patients with polimicrobial etiology.

For patients with VAP previous antibiotic usage and admission to a surgical unit were found to be independent risk factors for inadequate AB. Previous antibiotic exposure is one of the most evident risk factors for nosocomial infections due to antibiotic-resistant bacteria, and it was reported as an independent risk factor for inadequate AB in most of the previously published studies [6,10,22-24]. “Admission to a surgical ICU” as being a risk factor for inadequate AB is more difficult to explain. The separation of patients into medical and surgical units without identifying the performed surgical procedure may have affected the analysis of risk factors.

In our study cohort, all of the identified risk factors were shown to be indirectly associated with the acquisition of resistant bacteria for both VAP and HAP. However, in contrast to the results of other previously published studies, multivariate analysis showed that, presence of MDR bacteria was not an independent risk factor for both of the groups, [5,8]. This contradictory finding may be due to the MDR Acinetobacter spp. outbreak which had just begun early in the study period in our ICUs. This could also be a possible explanation of high rates of inadequate therapy in our study population in general. We reevaluated the empiric treatment choices after the antibiotic susceptibility profile of the epidemic clone had been identified. For some of the isolates colistin was the only drug of choice but since colistin was unavailable in the market, in Turkey at the time of the study, we were not able to use it as an empirical treatment option. As a result the presence of MDR bacteria was high in both of the treatment groups and the difference regarding the frequency of MDR bacteria between groups did not reach statistical significance. In patients with HAP, presence of MDR bacteria was associated with inadequate AB but in the final multivariate model this was not significant. Although we have included both culture positive and culture negative patients in the risk factor analysis, the impact of presence of MDR bacteria did not change when the analysis was performed in culture proven patients only.

In this study, we were not able to find any statistically significant difference in mortality rates between patients who received inadequate initial AB and patients who received adequate initial AB for both HAP and VAP. Available studies evaluating the impact of empirical AB have produced conflicting results; some found positive correlation between adequate empirical AB and survival, whereas others did not [8-15]. These contradictory results are probably ascribable to differences in patients and pathogens responsible for pneumonia; since in the studies those found higher mortality rates when AB was inadequate, the patients who received inadequate AB were the ones with pneumonia caused by the most difficult to treat microorganisms [5,11]. In a prospective study of patients with late-onset VAP; it was reported that infection due to non-fermenting gram-negative bacilli was the most important predictor of in-hospital mortality [22]. These pathogens were also the most common isolates in our study. However, the frequency of non-fermenting gram-negative bacilli in the inadequate and adequate AB groups was not significantly different and this may have affected the survival results. Another reason may be the difference in the definition of adequate AB as in some studies adequate AB was defined as AB with a favorable clinical response and in others it is described as microbiological recovery of susceptible organisms to the empirical antibiotic regimen. In the present study we used both of these definitions; in culture positive patients the adequacy of antibiotics were defined according to the results of the antimicrobial susceptibility testing whereas in culture negative patients clinical response to the empiric antibiotic regimen was taken into consideration. However the assosciation between the adequacy of the initial AB and survival did not change when the analysis was performed in culture positive patients only for both VAP and HAP.

In our study cohort although we could not find a positive correlation between adequate initial antimicrobial therapy and survival, we showed a significant increase in ICU length of stay, duration of mechanical ventilation and a prolonged clinical resolution in the inadequate AB group for both VAP and HAP. Other researchers also reported an increased length of stay and increased duration of mechanical ventilation when initial AB was inadequate in comparison with adequate AB [6,10]. Only in one study evaluating the outcome of patients with VAP, length of ICU and hospital stays were found to be similar in patients who did and did not receive adequate initial AB [11]. For patients receiving adequate AB, it was reported that clinical resolution of pneumonia usually begins during the first several days of treatment [6,25]. Our results were correlated with the literature, besides we showed a significant prolongation of the clinical resolution when the initial antibiotic therapy was inadequate.

The present study has several limitations. Its retrospective design is a limitation, since it was not possible to conduct an accurate analysis of any changes in the empirical AB regimens. On the other hand, the retrospective feature could have been an advantage as the prescription of initial AB was not influenced by a prospective evaluation during which a large spectrum initial AB could have been chosen. Another limitation is the impact of specific pathogens on the adequacy of initial AB and survival could not be performed because stratification into smaller groups would complicate entry in the logistic regression model, as each group would have a very small number of cases.

## Conclusions

In the present study, late-onset pneumonia and APACHE II score at the onset of HAP were found to be independent risk factors for inadequate initial AB in patients with HAP whereas previous antibiotic usage and admission to a surgical unit were found to be associated with inadequate initial AB in VAP. Our data suggests that the risk factors for inadequate initial AB were indirectly associated with the acquisition of resistant bacteria for both VAP and HAP. Moreover, patients who received inadequate initial AB with VAP or HAP, had a longer duration of hospital stay and an increased clinical resolution time. Our findings support the importance of investigating the local ecology by active surveillance cultures and follow-up of susceptibility patterns to establish adequate empirical AB. Therefore, consideration should be given to the empirical use of a broader-spectrum antibiotic that has not previously been administered, especially for the coverage of resistant gram-negative bacteria in order to minimize the occurrence of inadequate AB.

## Competing interests

The authors declare that they have no competing interests.

## Authors’ contributions

NP participated in study design and in data collection and interpretation, performed the statistical analysis and drafted the manuscript. HA and NO participated in study design, data collection and interpretation. DA participated in study design and revised the manuscript for important intellectual content. FC participated in data collection and interpretation. FK performed the statistical analysis and helped to draft the manuscript. GC revised the manuscript for important intellectual content. All authors read and approved the final manuscript.

## Pre-publication history

The pre-publication history for this paper can be accessed here:

http://www.biomedcentral.com/1471-2334/12/268/prepub
